# Insights into the molecular basis of long-term storage and survival of sperm in the honeybee (*Apis mellifera*)

**DOI:** 10.1038/srep40236

**Published:** 2017-01-16

**Authors:** Ellen Paynter, A. Harvey Millar, Mat Welch, Barbara Baer-Imhoof, Danyang Cao, Boris Baer

**Affiliations:** 1 Centre for Integrative Bee Research (CIBER), Bayliss Building (M316), The University of Western Australia, 35 Stirling Highway, 6009 Crawley, Western Australia; 2 ARC Centre of Excellence in Plant Energy Biology, Bayliss Building (M316), The University of Western Australia, 35 Stirling Highway, 6009 Crawley, Western Australia; 3 School of Animal Biology, 35 Stirling Highway, 6009 Crawley, The University of Western Australia, Western Australia

## Abstract

Honeybee males produce ejaculates consisting of large numbers of high quality sperm. Because queens never re-mate after a single mating episode early in life, sperm are stored in a specialised organ for years but the proximate mechanisms underlying this key physiological adaptation are unknown. We quantified energy metabolism in honeybee sperm and show that the glycolytic metabolite glyceraldehyde-3-phosphate (GA3P) is a key substrate for honeybee sperm survival and energy production. This reliance on non-aerobic energy metabolism in stored sperm was further supported by our findings of very low levels of oxygen inside the spermatheca. Expression of *GA3P dehydrogenase (GAPDH)*, the enzyme involved in catabolism of GA3P, was significantly higher in stored compared to ejaculated sperm. Therefore, long-term sperm storage seems facilitated by the maintenance of non-aerobic energy production, the need for only the ATP-producing steps of glycolysis and by avoiding sperm damage resulting from ROS production. We also confirm that honeybee sperm is capable of aerobic metabolism, which predominates in ejaculated sperm while they compete for access to the spermatheca, but is suppressed during storage. Consequently, the remarkable reproductive traits of honeybees are proximately achieved by differential usage of energy production pathways to maximise competitiveness and minimise damage of sperm.

The eusocial ants, bees and wasps offer unique opportunities to identify and study the molecular determinants responsible for exceptional levels of sperm quality and survival that have been reported in these species^1^,^2^,^3^,^4^,^5^,^6^,^7^. The reproductive females of social insects are typically referred to as queens and only mate at the beginning of their life, when they store sperm in a special organ referred to as the spermatheca^8^,^9^,^10^. Although queens never re-mate after they have started to lay eggs, they are capable of building colonies that survive for decades and consist of millions of individuals in some species^2^. Because queens are ultimately sperm limited, they are required to initially store large numbers of sperm and use them over prolonged periods of time to economically fertilise large numbers of eggs^11^,^12^,^13^. Such exceptional demands on sperm number and quality also impact social insect males, and some are known to produce exceptionally large ejaculates containing sperm of maximal quality^14^,^15^.

The details of how such remarkable reproductive traits are realised through the molecular functions of sperm proteins and the abundance and use of metabolites has not been studied in great detail. Storage for years to decades requires sperm to produce energy for maintenance and repair in order to maintain its full fertilisation potential. Such physiological activities are expected to trade-off with costs, for example damage by reactive oxygen species (ROS) associated with ATP production that will accelerate sperm senescence. Consequently, social insects are expected to have evolved physiological adaptations that minimise costs and maximise benefits to ensure long-term sperm survival. Here, we used an integrative -omics approach, to compare the underlying biochemical pathways used by ejaculated and stored sperm.

Previous studies indicate that biochemical pathways linked to energy metabolism are key contributors to the specific sperm traits reported in most social insects^16^,^17^. For example, proteins involved in glycolysis are significantly enriched in honeybee sperm compared with sperm of fruit flies or humans^18^. Furthermore, protein abundance differs between ejaculated and stored honeybee sperm, including a number of glycolytic enzymes, providing further evidence that energy metabolism is key for their long-term survival^17^. Glycolytic enzymes such as glyceraldehyde-3-phosphate dehydrogenase (GAPDH) and enolase, were more abundant in stored sperm but had a lower activity, suggesting wear and tear during storage^17^. Finally, sperm cells are surrounded by different glandular secretions in the ejaculation and storage phases; known as male-derived seminal fluid^19^ or female-derived spermathecal fluid^20^. Although both secretions contain energy metabolizing enzymes, the proteomes of these secretions differ quite substantially from each other. In honeybees, glycolytic enzymes are prominent in the female-derived spermathecal fluid but absent in male-derived seminal fluid^20^.

Based on these earlier studies, we quantified the effects of different substrates on energy metabolism, sperm physiology and survival. Here we directly measured sperm respiratory and glycolytic rates, transcript profiles and *in situ* oxygen concentrations. We identified glyceraldehyde-3-phosphate (GA3P) as a key substrate for energy production and survival in honeybee sperm. We found that this substrate keeps sperm alive during storage, is rapidly catabolised and provides the most efficient source of ATP for sperm function of all substrates tested. The relative mRNA levels of *GAPDH*, which encodes the enzyme catabolising GA3P were significantly higher in stored sperm compared to ejaculated sperm implying that sperm increase usage of GA3P for energy production during storage and maintain this capacity through translational processes. ATP production during storage using GA3P was also shown to be independent of oxygen, which minimises ROS-based damage to sperm. Oxygen concentrations inside the spermatheca were measured and shown to be substantially lower than in other honeybee tissues investigated.

## Results

### Metabolic activity of ejaculated sperm

**Figure 1:**
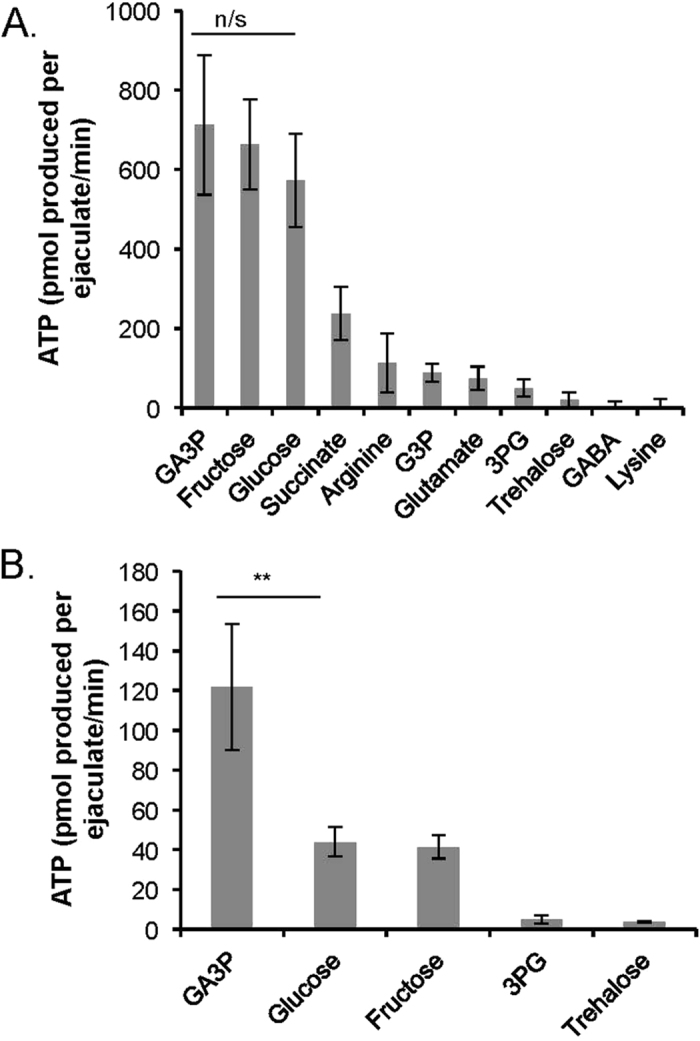
ATP produced from substrate metabolism in ejaculated sperm. ATP produced by (**A**) aerobic metabolism and (**B**) acidifying glycolytic metabolism of substrates. Error bars denote 1SE. n/s: *p* ≥ 0.05, **p ≤ 0.01. GA3P, glyceraldehyde-3-phosphate; G3P, glycerol-3-phosphate; 3PG, 3-phosphoglycerate; GABA, gamma-aminobutyric acid.

**Figure 2:**
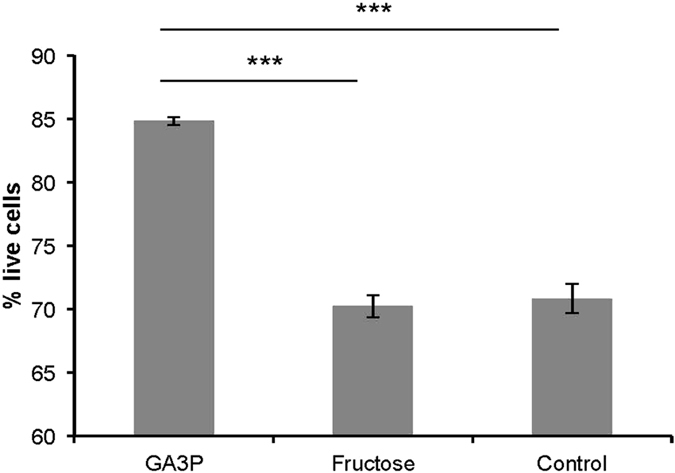
Sperm viability after 24 h incubation with different substrates. Error bars denote 1SE. ***p ≤ 0.001. GA3P, glyceraldehyde-3-phosphate.

To investigate the capacity for substrate metabolism using oxygen-dependent and oxygen-independent pathways in freshly ejaculated honeybee sperm, we carried out assays which measured the rate of aerobic metabolism, culminating in oxygen consumption, and acidifying glycolytic metabolism, culminating in acidification of the media surrounding sperm. Substrates for our assays were selected because they were previously reported to be metabolised by honeybee sperm or have been used in semen extenders. Our findings show that ejaculated honeybee sperm are capable of both metabolic pathways (Supplementary Fig. 1) and can utilise a number of different substrates in these processes. Furthermore, glucose, fructose and GA3P were used at substantially higher rates compared with other substrates, both via aerobic and acidifying glycolytic metabolism (Supplementary Fig. 1). These substrates also provided substantially higher ATP yields from aerobic metabolism compared to the remaining substrates (Fig. 1A). The ATP yields gained through aerobic metabolism were comparable for GA3P, glucose and fructose and did not differ significantly from each other (Mann-Whitney *U* test: *U* = 106.0; *n*_Glucose_ = 15, *n*_GA3P_ = 15; *p* = 0.787). However, GA3P use yielded a significantly higher ATP production via acidifying glycolytic metabolism when compared to glucose or fructose use (Mann-Whitney nonparametric *U* test: *U* = 50.0; *n*_Glucose_ = 15, *n*_GA3P_ = 15; *p* = 0.013 and *U* = 53.0; *n*_Fructose_ = 15, *n*_GA3P_ = 15; *p* = 0.009, Fig. 1B). This increase in ATP production was driven by the higher rate of acidifying glycolytic rate for GA3P compared with glucose or fructose (Supplementary Fig. 1). Furthermore, glucose and fructose initially need to be catabolised through a pathway which includes two ATP-consuming steps, while GA3P enters glycolysis after these steps, and therefore provides a higher total ATP production per mole of 3-carbon substrates. When we independently quantified the effect of GA3P and fructose on sperm survival, we found that sperm viability was significantly higher in samples containing GA3P compared to fructose or the control treatment without any substrate (Wilcoxon Signed-Rank test; *n* = 10; *Z* = −2.934; *p* < 0.001, Fig. 2).

### Metabolic activity of stored sperm

**Figure 3:**
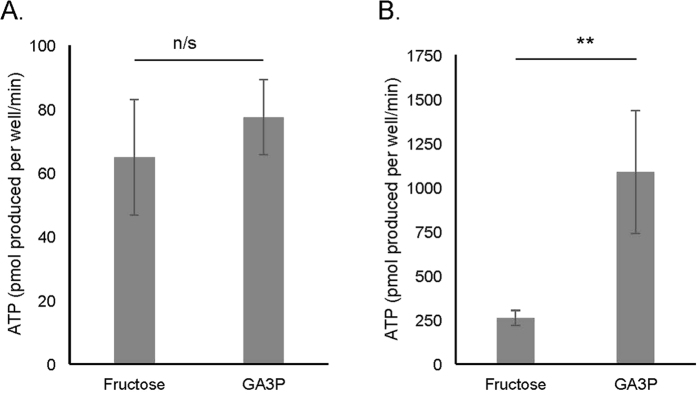
ATP produced from substrate metabolism in stored sperm. ATP produced by (**A**) aerobic metabolism and (**B**) acidifying glycolytic metabolism of substrates. Error bars denote 1SE. n/s: *p* ≥ 0.05, **p ≤ 0.01. GA3P, glyceraldehyde-3-phosphate.

Knowing that honeybee sperm are capable of metabolising a number of substrates, we quantified differences in metabolic usage of key substrates using stored sperm collected from the spermatheca of egg-laying queens. Using the aerobic pathway, stored sperm were capable of catabolising GA3P and fructose (Supplementary Fig. 2). Total ATP yields were significantly higher for GA3P use compared to fructose use (Mann-Whitney *U* test: *U* = 0.0; *n*_Fructose_ = 4, *n*_GA3P_ = 6; *p* = 0.010, Fig. 3). This result was mainly driven through changes in acidifying glycolytic metabolism, because no significant difference in ATP production was found between GA3P and fructose use through aerobic metabolism leading to oxygen consumption (Mann-Whitney *U* test: *U* = 9.0; *n*_Fructose_ = 4, *n*_GA3P_ = 6; *p* = 0.610, Fig. 3A). As expected from our findings in ejaculated sperm, GA3P use provided significantly more ATP through acidifying glycolytic metabolism compared to fructose use (Mann-Whitney *U* test: *U* < 0.001; *n*_Fructose_ = 4, *n*_GA3P_ = 6; *p* = 0.010, Fig. 3B). We concluded from these data that GA3P use is a key pathway for ATP production in honeybee sperm.

**Figure 4:**
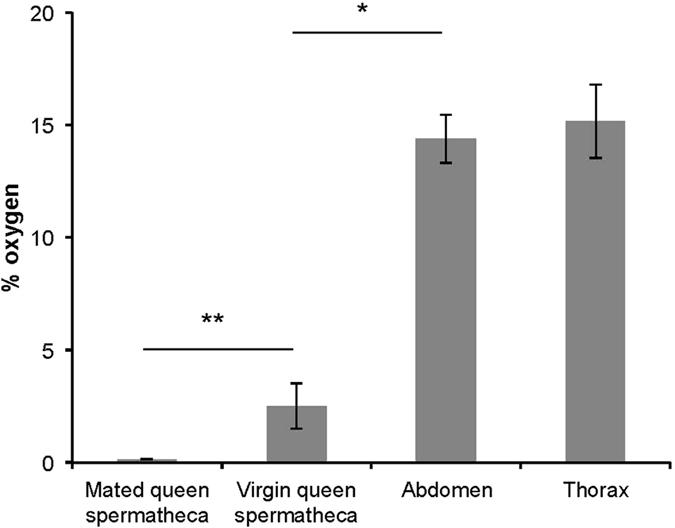
Oxygen concentrations in honeybee tissues. Error bars denote 1SE. *p ≤ 0.05, **p ≤ 0.01.

The potential importance of acidifying glycolysis using GA3P as a substrate for ATP production of stored sperm was also indicated by the oxygen concentration measurements obtained in different queen tissues (Fig. 4). Oxygen concentrations were approximately 6 times lower in the spermatheca compared to the abdomen or thorax of queens (Mann-Whitney *U* test: *U* < 0.001; *n*_Abdomen_ = 4, *n*_VQ_ = 4; *p* = 0.029). Furthermore, oxygen concentrations were approximately 16 times lower in the spermathecae of mated compared with virgin queens (Mann-Whitney *U* test: *U* < 0.001; *n*_VQ_ = 4, *n*_MQ_ = 7; *p* = 0.006, Fig. 4). These findings confirm a very low availability of oxygen to stored sperm, limiting their ability to produce ATP through aerobic metabolism. The metabolic measurements of stored sperm show that the cells are able to maximise ATP production under hypoxic conditions inside spermatheca using GA3P as a substrate.

### GAPDH production in sperm

To understand energy metabolism of stored and ejaculated sperm in more detail we compared relative expression levels of a number of genes including *GAPDH* (Table 1). We found that *GAPDH* transcripts were over 60 fold more abundant in stored sperm than in ejaculated sperm (Independent samples *t* test; *n* = 3, *t* = −7.946; *p* = 0.001). These data therefore provide a further indication that GAPDH, GA3P and the resulting acidifying glycolytic metabolism is a key contributor to long-term sperm survival.

## Discussion

**Figure 5:**
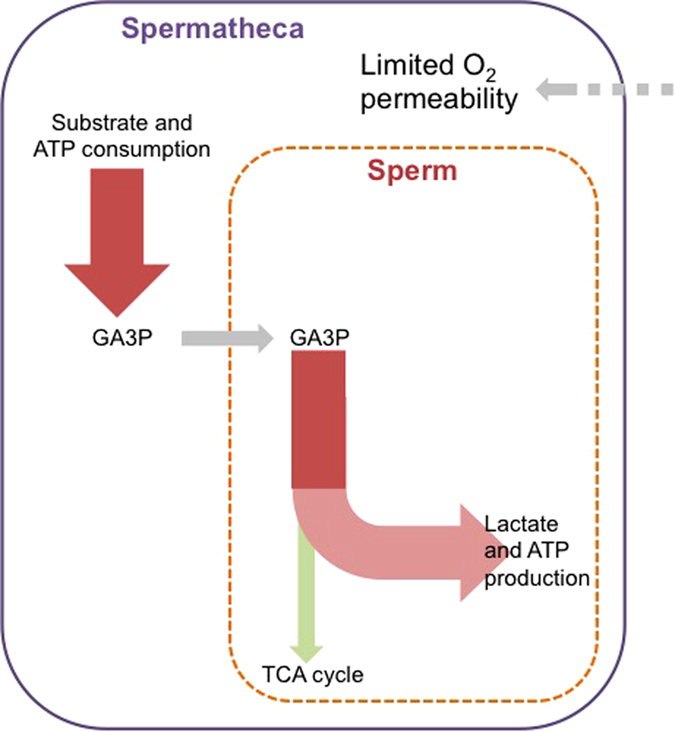
Proposed cooperative metabolism via acidifying glycolytic metabolism during sperm storage. GA3P, glyceraldehyde-3-phosphate.

We provide a number of novel insights into the unique physiological adaptations of honeybee sperm that we propose to underlie the remarkable ability of long-term storage and survival of social insect sperm (Fig. 5). Our findings indicate that honeybee sperm are able to maximise ATP production through aerobic metabolism and provide several lines of evidence that only part of the glycolytic pathway is predominantly responsible for the production of ATP in sperm stored inside the oxygen-deprived spermatheca. Our data provide strong indication that by using an oxygen-independent pathway, stored sperm may minimise ROS damage over the several-year long storage time. In the paragraphs below we discuss the broader implications of our findings for the reproduction and social lifestyle of honeybees.

We found that ejaculated sperm possess the complete biochemical machinery to produce ATP through aerobic metabolism. The availability of ATP during mating and prior to sperm storage is likely to be key for paternity success of honeybee sperm because queens are highly polyandrous and mate with many more males than required to ensure a complete filling of the spermatheca^9^. Although the filling of the spermatheca involves both active and passive processes, only 3–5% of sperm acquired during matings ultimately become stored and sperm and motility facilitates swimming up the spermathecal duct^21^. Flagellar movement of sperm is ATP-dependent, and energy production is thought to be of central importance for ejaculated sperm to outcompete rival sperm^22^. The use of aerobic metabolism during the process of sperm transfer/storage might therefore require the higher ATP production via aerobic metabolism during this competitive stage, resulting in physiological costs due to increased exposure to ROS. This implies trade-offs between sperm survival and competitiveness, which should be investigated in more detail in the future.

Our findings also allow provision of a detailed mechanism of those physiological processes that underlie long-term sperm survival in honeybees. With the arrival of sperm in the spermatheca, the conflicts over paternity are largely resolved^23^ because any further hostility between sperm compromises the fecundity of the queen, which is neither in the interest of queens nor her mates. Competition during egg fertilisation seems largely absent because honeybee queens only use very few sperm to fertilise their eggs, enabling them to maximise their lifetime fecundity in the absence of re-mating^13^. The physiology of stored sperm is therefore expected to change to maximise long-term survival and fertility. Here we show that stored sperm increases its capacity to produce ATP through acidifying glycolytic metabolism rather than aerobic metabolism. This finding is supported by our measurements of metabolic activity in stored sperm, and by the up-regulated gene expression levels of the enzyme metabolising GA3P. The presence of gene transcription in mature sperm has not been studied in any great detail, and sperm cells are thought to be transcriptionally silent. The observed presence of mRNA in sperm was proposed to be important for translation during capacitation or represent residual mRNA that is transferred to the oocyte during fertilisation^24^. However, several previous studies provided some evidence that sperm are able to transcribe mRNA^25^,^26^,^27^ and our data provide further empirical support for this idea, Our observations imply that sperm are transcriptionally active while stored inside the queen’s spermatheca to allow them to produce a selected group of proteins that they require to maximise survival during storage. A recent study confirmed that sperm enzymes involved in GA3P metabolism are prone to reduced activity during storage, consistent with wear and tear of enzymes in use, whereas enzymes involved in the initial steps of the glycolysis pathway were unaffected by storage^17^. Many of the glycolytic enzymes found in the spermathecal fluid also increase in abundance after mating, perhaps to partially counteract their reduced activity inside the sperm^17^. The high relative transcript level of *GAPDH* in stored sperm is consistent with the need for protein synthesis during storage. Sperm-specific isoforms of *GAPDH* have been identified in mammalian sperm, these have not been found in insect sperm^28^. However, it is possible that similar sperm-specific isoforms do exist in honey bee sperm which should be a subject for future study. ROS has been shown to reduce GAPDH activity in sperm from other species^28^. Although it appears that it may be possible to replace enzymes such as GAPDH during storage, the low oxygen environment found in the spermatheca is ideal for maintaining GAPDH function without ROS damage over a long period of time. Future research investigating metabolic changes in sperm over time following mating could provide further insight into this process of adaptation.

The low level of oxygen inside the spermatheca also implies avoidance of aerobic metabolism of stored sperm. Consequently, long-term sperm storage is not only achieved through specific adaptations related to energy production in sperm, but also by queens providing the sperm with an environment that is depleted of oxygen. Previous proteomic analysis revealed an increased abundance of glycolytic proteins in the spermathecal fluid after mating^20^. This may allow females to maintain the necessary machinery for the earlier steps of glycolysis to produce GA3P inside the spermatheca, which is then provided to sperm. As a consequence, queens provide sperm with a highly supportive environment and with a metabolic substrate that has a high ATP yield per mole of substrate (Fig. 5). These energetically costly queen contributions are expected to generate physiological consequences for the honeybee queen, such as trade-offs with immunity as seen in the leaf cutter ant *Atta colombica*^3^. The exact mechanism for oxygen depletion in the spermatheca is not known. However, there are several biochemical and morphological features that could contribute to maintaining an environment inside the spermatheca that is low in oxygen. It is known that the wall of the spermathecal is a thick sclerotized structure which could minimize influx of oxygen into the spermatheca^29^. Furthermore, the tracheal network surrounding the spermatheca may remove oxygen before it reaches the spermatheca^29^. Oxygen that does enter the spermatheca despite these morphological barriers might be removed by numerous oxidases present in the spermathecal fluid^18^,^20^. We conclude that long-term sperm storage is closely linked with energy metabolism of sperm and the avoidance of ROS damage, which is supported by findings of previous studies. For example, sperm and spermathecal fluid are enriched with antioxidant proteins^18^,^20^ and antioxidant protection of sperm within the spermatheca has previously been hypothesised to be important for honeybee sperm longevity^30^. Abundance of transcripts for antioxidant enzymes and enzymatic antioxidant activity are both higher in spermathecae of mated compared to virgin queens^30^,^31^ and sperm phospholipids are indeed shown to be protected from oxidation during storage^32^.

The insights we provided here of the molecular mechanisms that contribute towards the exceptional long survival of honeybee sperm have broader implications. Honeybees are key pollinators for more than 80 crops of agricultural interest, and are bred and managed to secure future crop pollination. Artificial insemination has become a key tool for the breeding of bee stock, and semen transport and storage is needed for such breeding activities. Our findings therefore offer new opportunities to maximise sperm survival and quality. For example, future research could investigate whether ejaculates kept in oxygen-free environments and provided with GA3P remain viable over prolonged periods of time, allowing the building of sperm banks of genetic bee stock similar to what is currently used for other domesticated animals. Further work could also test whether our approach to finding the optimal functional conditions in honeybees could also be applied to human sperm and could be used to assist *in vitro* fertilization.

## Methods

### Sperm sampling

All animals used for experiments were reared in an apiary at the University of Western Australia during spring and summer 2014. To collect ejaculates we used a method developed earlier^19^. In brief, sexually mature males (drones) were collected during early afternoons when they left their colonies to participate in their daily mating flights and anesthetized with chloroform to initiate ejaculation. To advance the ejaculatory process, males were squeezed between two fingers and semen eventually appearing at the tip of the male’s endophallus was collected with a 10 uL micropipette.

To collect stored sperm, we anaesthetised naturally-mated, egg-laying queens aged 9–18 months and dissected their spermathecae. We pierced a small hole in the spermatheca with an injection needle after removal of the tracheal network and used a glass capillary to remove sperm from the lumen as previously described^20^. All sperm samples were kept at room temperature during the short period between collection and assays.

### Sperm metabolic measurements

Single ejaculates, comprising sperm and seminal fluid were individually placed in 180 μL medium based on Dulbecco’s Modified Eagle Medium (DMEM: 1.8 mM calcium chloride, 0.8 mM magnesium chloride, 5.4 mM potassium chloride, 143 mM sodium chloride, 0.91 mM sodium phosphate monobasic, 40 μM phenol red, pH 7, hereafter DMEM Salts (DS)) in a well of a XF96 microplate (Seahorse Bioscience, North Billerica, USA). Two samples of stored sperm including spermathecal fluid, were combined into a single XF96 microplate well, as sperm numbers collected per spermatheca were much lower than those sampled per ejaculate. The microplate was centrifuged at 2000 × *g* for 20 min to ensure cells were fixed to the bottom of the wells, which was confirmed by microscopy. The assay was carried out using a XF96 Flux Analyser (Seahorse Bioscience) at hive temperature, 35 °C^33^. Samples were mixed for 210 seconds, followed by measurements for 300 seconds, which was repeated four times for each sample. To each tube, 20 μL of DS medium containing 50 mM of one of 11 substrates of interest was added, resulting in a final concentration of 5 mM. Substrates for the assays included glucose, fructose, trehalose, glyceraldehyde-3-phosphate (GA3P), glycerol-3-phosphate (G3P), 3-phosphoglycerate (3PG), glutamate, succinate, gamma-aminobutyric acid (GABA), arginine and lysine (Sigma-Aldrich, St. Louis, USA). Six biological replicates per substrate and assay were used, and a total of three independent assays were conducted for each. Consequently ~18 measurements became available for all of the substrates tested, which were run alongside six substrate-free controls per assay.

Oxygen consumption rate (OCR) and extracellular acidification rate (ECAR) were directly obtained from the XF96 Flux Analyser software version 1.0 (Seahorse Bioscience) using the first rate measurements following substrate injection. Buffer capacity for each compound was determined empirically according to the manufacturer’s instructions and were consequently used along with ECAR to calculate proton production rates (PPR). Aerobic metabolic rate was calculated using OCR, by calculating the moles of oxygen used during the catabolism of a mole of substrate. We used the term aerobic metabolism to refer to energy production resulting in oxygen consumption, which includes glycolysis and mitochondrial respiration. Acidifying glycolytic rate was similarly calculated using PPR which was first adjusted for non-glycolytic acidification by CO_2_. We used the term acidifying glycolysis to those biochemical steps that result in energy production through acidification but without oxygen consumption. We decided against the use of “lactic acid fermentation” because we have no direct evidence that lactate is the end product produced.

ATP yields were calculated based on the catabolism of one mole of each of the substrates tested, both via aerobic and acidifying glycolytic metabolism. This allowed calculation of the total ATP production for each sperm sample through aerobic and acidifying glycolytic metabolism based on the amount of ATP produced per mole of substrate per minute and the number of moles of substrate catabolised per minute (as above).

### Sperm viability assays

Sperm viability was quantified after completion of metabolic measurements as outlined above for 6 biological replicates per substrate by transferring samples into Eppendorf tubes to quantify sperm viability by counting the number of live and dead sperm under a fluorescent microscope as described previously^34^,^35^. Briefly, 5 μL of sample was placed on a microscope slide with 5 μL SYBR14 and incubated for 10 minutes at room temperature in the dark, followed by 1 μL propidium iodide and incubated for seven minutes prior to examination under the microscope. A minimum of 400 sperm from each sample was counted in triplicate and sperm viability calculated as the number of live sperm divided by the total number of sperm investigated. Data of the three technical replicates were averaged for statistical analyses. We found that sperm viability did not vary significantly between substrates, or as a result of handling during metabolic measurements (Supplementary Fig. 3).

To investigate how individual key substrates affect sperm viability, we collected single ejaculates as outlined above in 1.5 mL DS and gently mixed them by inversion. The sample was split into three 180 μL aliquots and fructose, GA3P or medium only (as a control) was added to a final concentration of 5 mM. Sperm samples were incubated in closed Eppendorf tubes at room temperature for 24 h and sperm viability was quantified afterwards as described above. Ten biological replicates became available per treatment and sperm viabilities were quantified twice per sample and averaged for statistical analyses.

### Tissue oxygen concentrations

To compare oxygen concentrations in different bee tissues, a calibrated TX3 trace oxygen microoptode (PreSens, Regensburg, Germany) encased in a needle-type housing was mounted onto a micro-manipulator. Once in place, the microoptode was carefully pushed out of the needle to expose the tip to the surrounding tissue, and remained there for two minutes until measurements had stabilised. After each measurement, the microoptode was rinsed thoroughly with water. For thorax measurements, three queens were decapitated and the needle inserted into the thorax through the arisen hole. Oxygen concentrations in the abdomen of four queens were measured by separating the abdomen from the thorax and inserting the microoptode needle through the consequent opening in the abdomen. Oxygen concentrations were also measured in the spermathecae of four mated and seven virgin queens. To do this we removed the tracheal network surrounding the spermatheca and inserted the needle into the spermathecal lumen.

### Transcriptomic analysis

RNA was extracted from stored and ejaculated sperm collected from 9 males and 9 queens as described above. Sperm was pooled in triplicate to produce three independent biological replicates for both stored and ejaculated sperm. RNA collected from whole lysates of 24 hour old larvae were used as a positive control for RNA extraction, cDNA synthesis and quantitative, real-time PCR. RNA isolations were carried out using TRIzol Reagent (Thermo Fisher Scientific, Waltham, USA) and 1 μL GenElute LPA (Sigma-Aldrich) following manufacturer’s instructions. Pellets were resuspended in 30 μL RNase-free water (Baxter, Deerfield, USA) and stored at −80 °C prior to further experiments.

RNA and cDNA samples were purified using a modified Serapure method^36^ and stored at −80 °C until required. DNase treatments were carried out using RQ1 RNAse-free DNase (Promega, Madison, USA) according to manufacturer’s instructions. Quantification was carried out using Qubit™ RNA assays (Life Technologies) and a Qubit 2.0 Fluorimeter (as per manual).

From larval samples, 500 ng of DNase-treated RNA was used for cDNA synthesis. As the amount of RNA isolated from sperm samples was typically below the level of detection, half of each sample was used for cDNA synthesis, and half was used as a negative control. DNase-treated template RNA was reverse transcribed using M-MLV RT Reverse Transcriptase (Promega) as per manufacturer’s instructions, then stored at −80 °C until required.

Primers specific for each transcript (Supplementary Table 1) of interest were designed using Primer-BLAST (www.ncbi.nlm.nih.gov/tools/primer-blast). Products were amplified from larval cDNA using Kapa HiFi HotStart DNA Polymerase PCR Kit (Kapa Biosystems, Wilmington, USA) and specificity was confirmed by direct sequencing of PCR products. To validate qPCR amplicons generated with these primers, reactions were set up using SYBR Fast qPCR Master Mix (Kapa Biosystems) and melt curves were generated via qPCR using Lightcycler 480 (Roche, Basel, Switzerland) to confirm unique target amplification. A dilution series using PCR products was created for each gene of interest to create a standard curve, and Crossing Point Cycle Values of gene expression values obtained from the cDNA used to calculate relative abundance. Actin was used as reference gene for normalisation between samples using primers designed previously^37^.

### Data analysis

Statistical analyses were carried out using SPSS version 21 (IBM, Armonk, USA). Because OCR and ECAR data were not normally distributed, non-parametric Kruskal-Wallis and Mann-Whitney *U* tests were used to test for statistical differences between substrates. To statistically analyse 24 h sperm viability comparisons we used a Friedman’s ANOVA. Wilcoxon Signed-Rank tests were used to test for differences between substrates. Student’s *t* tests for independent samples were used to statistically compare changes in gene expression.

## Additional Information

**How to cite this article:** Paynter, E. *et al*. Insights into the molecular basis of long-term storage and survival of sperm in the honeybee (*Apis mellifera*). *Sci. Rep.* **7**, 40236; doi: 10.1038/srep40236 (2017).

**Publisher's note:** Springer Nature remains neutral with regard to jurisdictional claims in published maps and institutional affiliations.

## Supplementary Material

Supplemental Information

## Figures and Tables

**Table 1:** Relative mRNA abundance of a various metabolic enzymes in stored and ejaculated sperm.

Protein	Ratio stored/ejaculated
Glyceraldehyde-3-phosphate dehydrogenase 2	61***
Aldose reductase	6.9**
Aspartate aminotransferase	5.6*
Phosphate carrier protein	4.0*
Fumarate hydratase	2.0
Hexokinase type 2	0.8
Cytochrome c	0.4
Bifunctional ATP-dependent dihydroxyacetone kinase	0.4
Ornithine aminotransferase	0.1

^***^p ≤ 0.001, ^**^p ≤ 0.01, ^*^p ≤ 0.05.
