# Supplementary material for: Insights into the molecular basis of long-term storage and survival of sperm in the honeybee (Apis mellifera)
Source: Sci Rep. 2017 Jan 16;7:40236. doi: 10.1038/srep40236 (PMC5238380; doi:10.1038/srep40236)

# Insights into the molecular basis of long-term storage and survival of sperm in the honeybee (*Apis mellifera*)

**Authors:**

Ellen Paynter [1,2], . Harvey Millar [1,2], Mat Welch [1], Barbara Baer-Imhoof [1], Danyang Cao [1], Boris Baer [1,2 *]

[1] Centre for Integrative Bee Research (CIBER), Bayliss Building (M316), The University of Western Australia, 35 Stirling Highway, 6009 Crawley, Western Australia

[2] ARC Centre of Excellence in Plant Energy Biology, Bayliss Building (M316), The University of Western Australia, 35 Stirling Highway, 6009 Crawley, Western Australia

***Corresponding Author**

Prof Boris Baer

Centre for Integrative Bee Research (CIBER), Bayliss Building (M316), the University of Western Australia, 35 Stirling Highway, 6009, Crawley, Western Australia

E-Mail: boris.baer@uwa.edu.au

Tel: +61 8 6488 4495

**Supplementary figure legends**

**Supplementary Figure 1.** Graph showing substrate-dependant aerobic metabolism and acidifying glycolytic metabolism of single honeybee ejaculates when incubated with different substrates. Error bars denote 1SE. GABA, gamma-aminobutyric acid; GA3P, glyceraldehyde-3-phosphate; G3P, glycerol-3-phosphate; 3PG, 3-phosphoglycerate.

**Supplementary Figure 2.** Substrate-dependant (A) aerobic metabolism and (B) acidifying glycolytic metabolism of honeybee sperm dissected from spermathecae when incubated with different substrates. Error bars denote 1SE. GA3P, glyceraldehyde-3-phosphate.

**Supplementary Figure 3.** Sperm viability following metabolic measurements of (A) ejaculated sperm and (B) sperm dissected from spermathecae. GA3P, glyceraldehyde-3-phosphate.

# Supplemental Figure 1

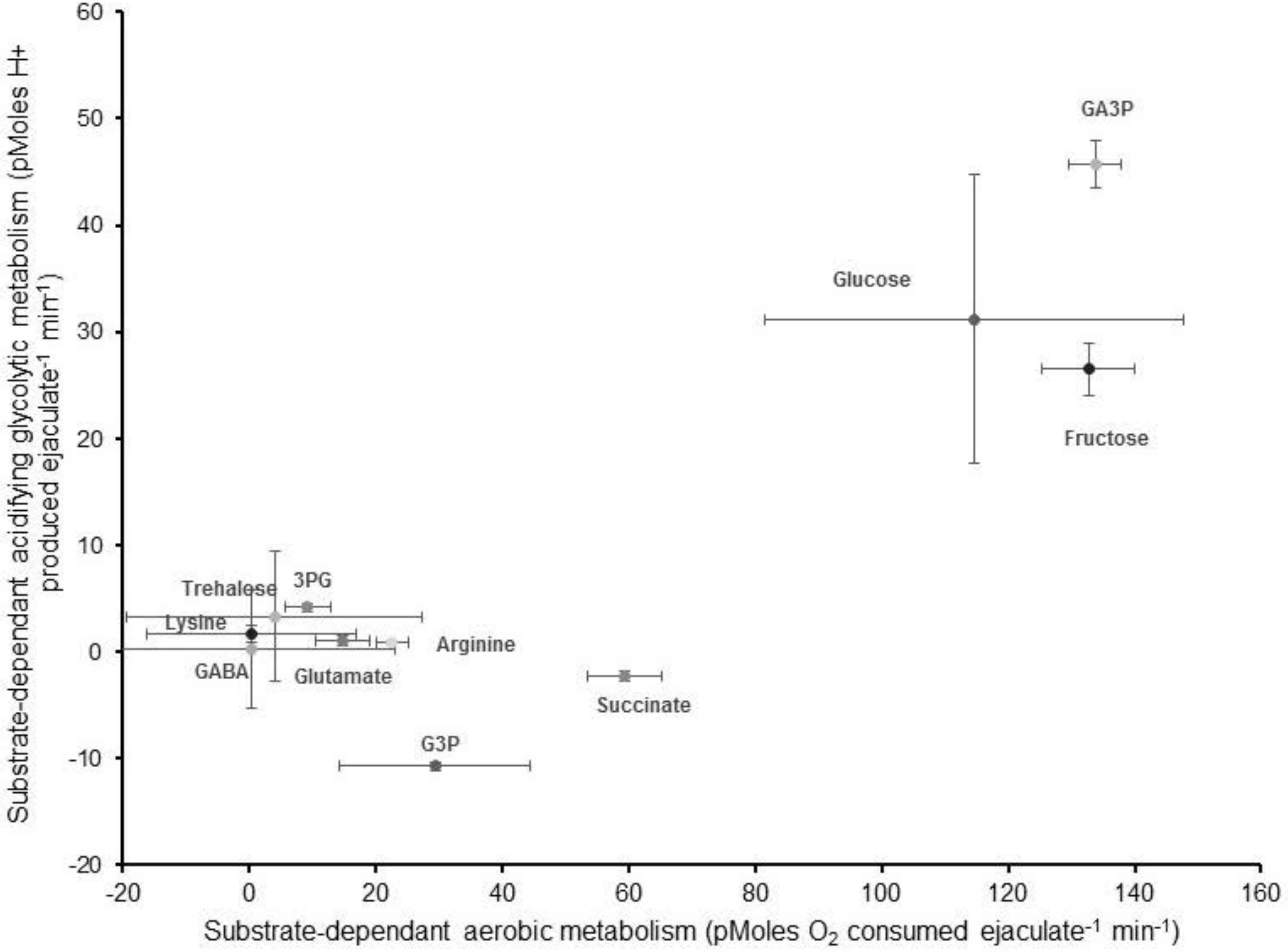

# Supplemental Figure 2

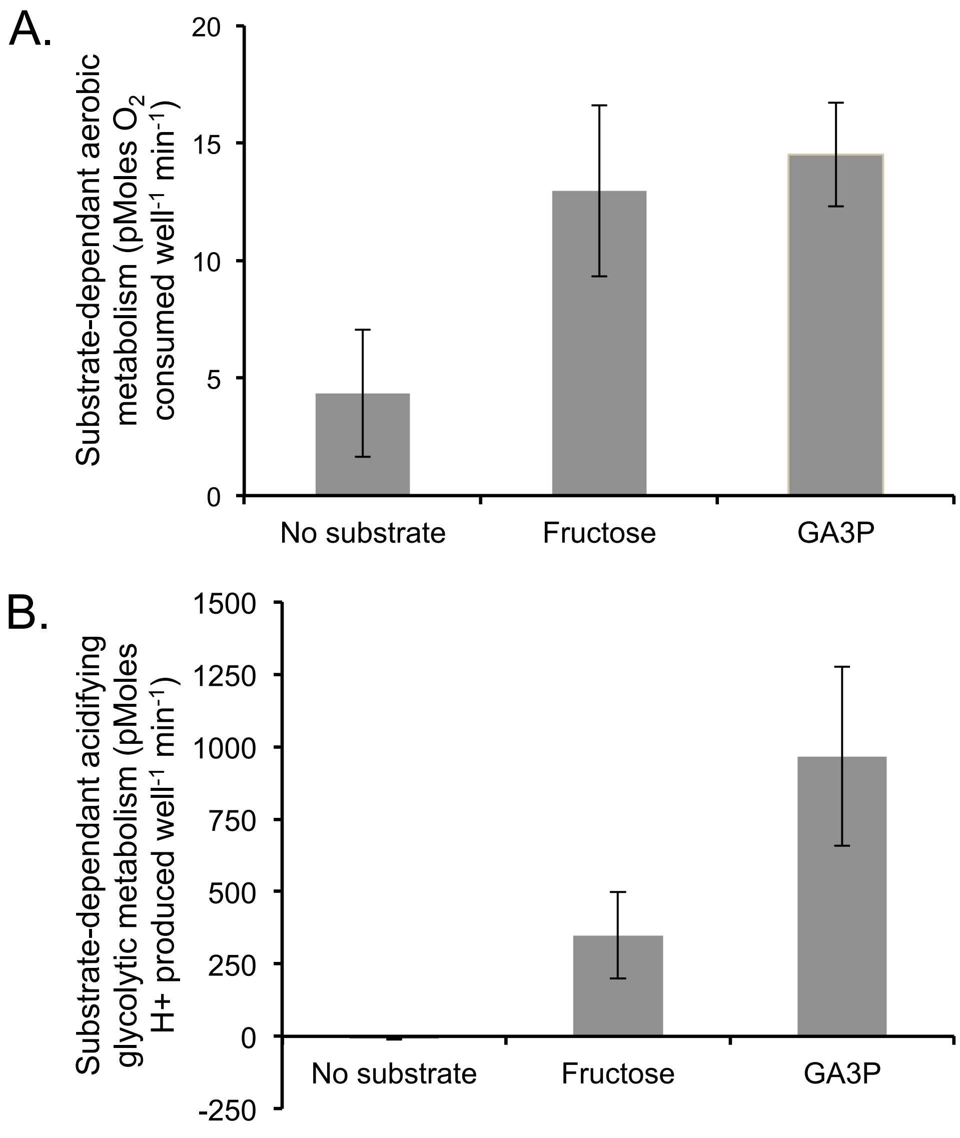

# Supplemental Figure 3

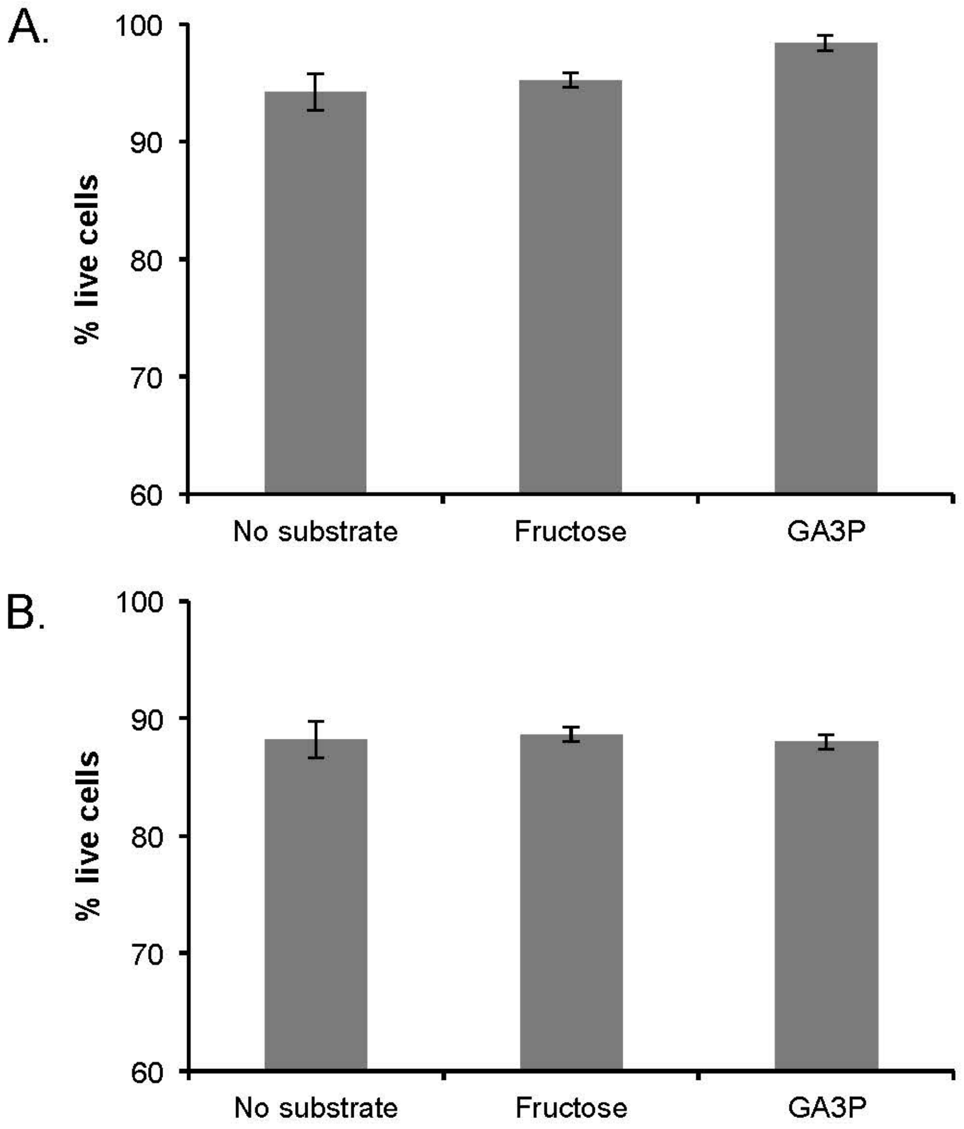

Supplement: Supplemental Information [file srep40236-s1.pdf]
